# THE BOSTON HOSPITAL-IN-HOME PROGRAM—A LOCAL PERSPECTIVE

**DOI:** 10.1093/geroni/igad104.0536

**Published:** 2023-12-21

**Authors:** Shivani Jindal, Brittany Pietruszka, Rachel Meears, James Parris

**Affiliations:** VA Boston Healthcare System, Boston, Massachusetts, United States; VA Boston Healthcare System, Boston, Massachusetts, United States; VA Boston Healthcare System, Boston, Massachusetts, United States; VA Boston Healthcare System, Boston, Massachusetts, United States

## Abstract

The Hospital-in-Home (HIH) model provides patient centered care, may reduce complications and costs. VA Boston Healthcare System (VABHS) planned its HIH program in 2018 and enrolled its first patient in May 2019. HIH provided care to 45 patients in 2019 which grew to 185 in 2022. The team is comprised of 2 clinicians (a hospitalist physician and a physician assistant), a nurse manager, 5 registered nurses (RN), a social worker and a pharmacist. The program practices complementary HIH (admissions from inpatient care) and substitutive care (all other admissions). At this time, majority of the recruitment comes from the inpatient services, followed by heart failure and primary care clinics. The program catchment area is about 25 miles from the West Roxbury campus of VABHS. A hybrid visit approach is taken for clinician visits - initially, the veteran is seen in person or by video by the clinician on admission to the program. Subsequent visits are done with RN assessments in the home and the clinician conducting the visit by telephone or video. In FY 2021, conditions of patients treated by HIH include congestive heart failure (50%), chronic obstructive pulmonary disease (14%), wound care (6%), diabetes (2%) and other conditions (20%). In FY 2022, the average daily census was 15.9 veterans and average visits per day 4.8. The HIH program outcomes include reduction of bed days of acute care in the hospital and reduction in the use of unnecessary medications through deprescribing which may translate to cost avoidance for the medical center.

